# Gut microbial ammonia as a mediator of PFOS neurotoxicity and its remediation by the flavonoid Icaritin

**DOI:** 10.1080/19490976.2026.2620125

**Published:** 2026-02-02

**Authors:** Yang Yi, Wenfang Zhang, Yu Wei, Wang Ran, Dongjing Liu, Weikun Deng, Songyuan Duan, Jiyong Yao, Lianhang Wang, Yuandong Zhang, Jianmei Gao, Qihai Gong

**Affiliations:** a Key Laboratory of Basic Pharmacology of Ministry of Education and Joint International Research Laboratory of Ethnomedicine of Ministry of Education, Zunyi Medical University, Zunyi, People's Republic of China; b Department of Pharmacology, Key Laboratory of Basic Pharmacology of Guizhou Province and School of Pharmacy, Zunyi Medical University, Zunyi, Guizhou, People's Republic of China; c Department of Pharmacology, Guizhou Medical University, Guiyang, People's Republic of China

**Keywords:** microbiota-gut-brain axis, perfluorooctane sulfonate, icaritin, cognitive dysfunction, ammonia metabolism

## Abstract

Perfluorooctane sulfonate (PFOS), a persistent environmental pollutant, is associated with cognitive dysfunction through mechanisms involving neuroinflammation, oxidative stress, and metabolic disruption. Icaritin, a bioactive flavonoid with antioxidant and anti-inflammatory properties, exhibits therapeutic potential, though its efficacy against PFOS-induced cognitive impairment remains unexplored. Herein, a mouse model of PFOS-induced cognitive dysfunction was established and treated with oral ICT. Integrated 16S rRNA sequencing and untargeted metabolomics revealed that ICT restored gut microbial homeostasis by enriching beneficial genera (e.g. *Akkermansia*, *Lactobacillus*) and reducing ammonia-producing bacteria (e.g. *Proteus*, *Helicobacter*, *Escherichia*), thereby improving gut barrier integrity. Metabolomic profiling identified significant perturbations in ammonia-related pathways, particularly arginine and proline metabolism, underscoring ammonia dysmetabolism as a pivotal mediator of PFOS neurotoxicity. These modifications attenuated systemic and cerebral ammonia accumulation, mitigated neuroinflammation and oxidative stress, and ultimately improved cognitive function. Our findings elucidate ammonia dysmetabolism as a central mechanism in PFOS-induced cognitive decline and highlight the microbiota–gut–brain axis as a promising therapeutic target. This study provides a mechanistic foundation for targeting microbial and metabolic pathways in environmental neurotoxicity.

## 1. Introduction

Perfluorooctanesulfonic acid (PFOS), a prominent per- and polyfluoroalkyl substance (PFAS), is widely used in industrial and consumer products such as firefighting foams, textiles, and food packaging owing to its exceptional stability, hydrophobicity, and oleophobicity. These properties contribute to its environmental persistence and widespread dissemination through water, air, and soil.^1-3^ Despite regulatory restrictions in many countries, PFOS continues to bioaccumulate, posing serious risks to ecosystems and human health.^4^ Exposure to PFOS has been linked to diverse adverse effects including metabolic dysregulation, immunotoxicity, neurotoxicity, and potential carcinogenicity.^5^^,^^6^ A growing body of evidence further indicates that PFOS impairs cognitive function, particularly in vulnerable populations such as children and the elderly.^7-9^

Cognitive dysfunction involves deficits in attention, memory, and executive function, driven by mechanisms involving neuroinflammation, oxidative stress, and impairments in synaptic plasticity.^10-13^ PFOS exacerbates these pathways by crossing the blood–brain barrier, promoting cerebral oxidative stress and neuroinflammation, and ultimately impairing learning and memory.^7^^,^^14^ Recent studies highlight the contribution of metabolic disturbances to cognitive decline.^15^^,^^16^ In particular, ammonia, which is a neurotoxic metabolite of nitrogen metabolism, has become a focus of investigation.^17^^,^^18^ The human body produces approximately 1,000 mmol of ammonia from amino acid catabolism, maintaining systemic levels below 50 μM primarily via the hepatic urea cycle.^19^ Ammonia exists in equilibrium between ammonium ions (NH_4_^+^) and gaseous NH_3_, the latter readily diffusing across cellular membranes and the blood–brain barrier.^20^^,^^21^ The brain is highly vulnerable to ammonia toxicity due to its lack of a complete urea cycle.^22^^,^^23^ Astrocytic glutamine synthetase (GS) serves as the primary detoxification mechanism, converting ammonia and glutamate into glutamine (Gln).^24^^,^^25^ Gln in turn supports neurotransmitter synthesis such as glutamate and *γ*-aminobutyric acid (GABA), and its deficiency has been implicated in various neurological disorders such as Alzheimer’s disease, temporal lobe epilepsy, Parkinson’s disease, and depression.^26^^,^^27^ Although the neurotoxicity of ammonia is well-documented, its physiological roles remain poorly understood. Additionally, gut microbiota dysbiosis has emerged as a key contributor to cognitive impairment, though underlying mechanisms remain elusive.^28^^,^^29^ This study proposes ammonia as a gut–brain signaling molecule and investigates its role in linking gut microbiota metabolism to PFOS-induced cognitive decline.

Natural products, particularly those derived from traditional Chinese medicine, have attracted interest for their multifunctional bioactivities and favorable safety profiles.^30^^,^^31^ Icaritin (ICT), a flavonoid compound derived from Herba *Epimedii*, and approved use in hepatocellular carcinoma, demonstrates notable neuroprotective, antioxidant, and anti-inflammatory effects.^32-36^ Its potential in treating nervous system disorders warrants further exploration.

Here, we systematically evaluated whether ICT mitigates PFOS-induced cognitive dysfunction and examined the involvement of the gut microbiota using multi-omics and fecal microbiota transplantation (FMT). Our results demonstrate that ICT alleviated PFOS-induced cognitive deficits by modulating the microbiota–gut–brain axis, reducing ammonia accumulation in circulation and the brain, and reestablishing ammonia metabolic homeostasis. This restoration enhances astrocytic Gln synthesis, attenuates neuroinflammation and oxidative stress, and ultimately improves cognitive outcomes. These findings not only elucidate the therapeutic potential of ICT but also validate the gut microbiome as a promising target for treating environmental toxicant-induced cognitive impairment.

## 2. Materials and methods

### 2.1. Animals

A total of male ICR mice (7–8 weeks old, 28–32 g) was obtained by Hunan SJA Laboratory Animal Co., Ltd. (Hunan, China; Certification No. SYXK (Qian) 2021-0003). The animals were acclimatized under specific pathogen‐free (SPF) conditions at a temperature of 22–24 °C and 45%–55% relative humidity, with a 12 h/12 h light/dark cycle, housed five mice per cage. All experimental procedures were performed in compliance with the Guidelines for the Care and Use of Laboratory Animals published by the National Institutes of Health (NIH Publication no. 8023, revised 1978), and were approved by the Ethics Committee of Zunyi Medical University (Approval No. ZMU21-2409-020).

### 2.2. Materials

**Figure 1.**
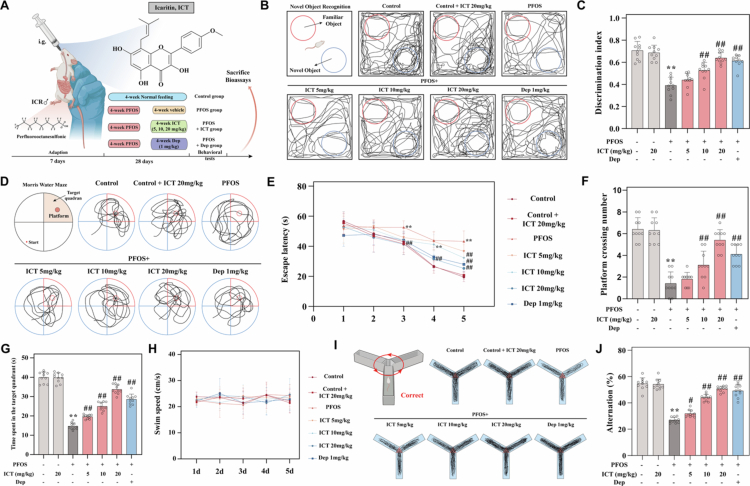
ICT alleviates cognitive dysfunction induced by PFOS in mice. The experimental timeline (A) illustrates the 28-d exposure period wherein mice received daily oral administration of PFOS (50 mg/kg) concurrently with ICT (5, 10, or 20 mg/kg) or donepezil (1 mg/kg), followed by sequential behavioral evaluations. (B) Representative search trajectories of mice in the novel object recognition (NOR) test. (C) Quantitative analyzes of discrimination index in NOR. (D) Representative search trajectories of mice in the MWM test. (E) Escape latency during the 5-day training period, showing daily learning curves. (F) Number of platform crossings during the probe trial, indicating spatial memory retention. (G) Time spent in the target quadrant during the probe trial, reflecting spatial reference memory. (H) Swimming speed of mice on the test day in the MWM test, demonstrating no motor impairment affecting cognitive performance. (I) Representative movement trajectory of mice in the Y-maze test. (J) Corresponding calculated spontaneous alternation rate, measuring working memory performance. The data were presented as the mean ± SEM (*n* = 10). ^**^P < 0.01 *vs.* Control group; ^#^*P* < 0.05, ^##^*P* < 0.01 *vs.* PFOS group.

ICT (purity ≥ 98%, CAS.118525-40-9, Figure 1A) was obtained from Shanghai Renjie Biotechnology Co., Ltd (Shanghai, China). PFOS (purity ≥ 98%, Figure 1A) and Donepezil were purchased from Sigma-Aldrich., Ltd (USA). The Alcian Blue-Periodic Acid-Schiff (AB-PAS) staining kit (G1285) was sourced from Solarbio science & technology Co., Ltd (Beijing, China). ELIZA kits for interleukin-1β (IL-1β, RJ16944), interleukin-6 (IL-6, RJ16958), and tumor necrosis factor-*α* (TNF-*α*, RJ17929), reactive oxygen species (ROS, RJ17213), malondialdehyde (MDA, RJ16984), catalase (CAT, RJ17177), superoxide dismutase (SOD, RJ17004), glutathione peroxidase (GSH-Px, RJ17154), glutamine synthetase (GS, RJ25744), glutamine (Gln, RJ17148), ammonia (NH3, RJ12779), and carbamoyl-phosphate synthase 1 (CPS-1, RJ21487) were acquired from Shanghai Renjie Bioengineering Institute (Shanghai, China). GFAP (16825-1-AP), C3 (21337-1-AP), S100A10 (11250-1-AP) and *β*-tubulin (10094-1-AP) were purchased from Proteintech (Wuhan, China). *β*-amyloid (Aβ_1-42_) peptide was purchased from Macklin (Shanghai, China). Primary antibodies against ZO-1 (ab276131), Claudin-1 (ab307692), Occludin (ab216327), *β*-actin (ab8227) were acquired from Abcam (Cambridge, UK). Ampicillin (69-53-4), Vancomycin (1404-90-6), Neomycin sulfate (1405-10-3), Metronidazole (443-48-1), and Lactulose (4618-18-2) were acquired from MedChem Express (Monmouth Junction, NJ, USA).

### 2.3. Treatments and sample collection

Experiment 1: To evaluate the therapeutic potential of ICT on PFOS-induced cognitive dysfunction, ICR mice were acclimatized for one week and randomly allocated into seven groups (*n* = 10): Control, Control + ICT (20 mg/kg), PFOS, PFOS + ICT (5 mg/kg), PFOS + ICT (10 mg/kg), PFOS + ICT (20 mg/kg), and PFOS + donepezil (Dep, 1 mg/kg). The Control and Control + ICT groups received an equivalent volume of saline. All other groups were administered PFOS (50 mg/kg) via gavage once daily to induce cognitive impairment. Concurrently, treatment groups received corresponding doses of ICT or donepezil once daily for 28 consecutive d. At the end of the treatment period, fresh fecal samples were aseptically collected for 16S rRNA analysis. Behavioral tests were subsequently conducted to assess cognitive function. Then, animals were euthanized with sodium pentobarbital (50 mg/kg, i.p.) for the collection of blood and then quickly sacrificed by cervical dislocation for sample collection (as outlined in Figure 1A). Blood samples were centrifuged to isolate serum, which was stored at −80 °C. Brain and ileum tissues were carefully dissected, with one portion fixed in 10% formalin for histopathological evaluation, and the remainder flash-frozen for storage at −80 °C for subsequent analysis.

Experiment 2: To elucidate whether the gut microbiota mediates the protective effects of ICT against PFOS-induced cognitive impairment, a FMT approach was implemented. Mice were randomly assigned into three groups (*n* = 10): Control, PFOS, and PFOS + FMT. After a 7-day acclimation period, all of mice received an antibiotic cocktail (ABX) consisting of ampicillin, vancomycin, neomycin sulfate, and metronidazole (each at 1 g/L) ad libitum in drinking water for one week to deplete gut microbiota and establish a pseudo-germ-free model. Fecal donors consisted of mice that received 28-day oral treatment with PFOS and ICT (20 mg/kg). Donor mice were orally treated with ICT (20 mg/kg) for 28 d, and fresh fecal samples were collected from donor mice 48 hours after the final ICT gavage. This interval was implemented to allow for drug clearance. The samples were then homogenized and prepared as suspensions for transplantation. Recipient mice were administered 0.2 mL of fecal suspension via daily gavage for 28 consecutive d. After FMT, fecal samples were collected for microbial analysis, and cognitive behavior was assessed, and blood, brain, and ileal tissues were collected and processed as described in Experiment 1.

Experiment 3: To assess the role of ICT in modulating gut ammonia metabolism in the context of PFOS-induced cognitive dysfunction, mice were allocated into three experimental groups (*n* = 10): Control, PFOS, and PFOS + lactulose (LAC). The control group received an equivalent volume of saline via daily gavage. While both the PFOS and PFOS + LAC groups were administered PFOS to induce cognitive impairment. The PFOS + LAC group was additionally received LAC (5 g/kg) orally once daily for 28 d (as schematized in Figure 7A). At the endpoint, fresh fecal samples were collected and stored at −80 °C for further biochemical and molecular analyzes.

### 2.4. Statistical analysis

All quantitative data are presented as the mean ± SEM from at least five to ten independent replicates. Comparisons among three or more groups were performed using one-way analysis of variance (ANOVA), followed by post hoc tests: Bonferroni’s test was applied when homogeneity of variance was confirmed, and Dunnett’s T3 test was used in cases of unequal variance. All statistical analyzes were conducted using SPSS software (version 29.0; SPSS Inc., Chicago, IL, USA). A probability value of *P* < 0.05 was considered statistically significant.

Other detailed experimental methods, including behavioral tests, histopathological assessment, AB-PAS Staining, immunofluorescent staining, enzyme-linked immunosorbent assay, 16S rRNA Sequencing, untargeted metabolomics, FMT, and Western blot, were provided in the Supplementary Materials.

## 3. Results

### 3.1. ICT mitigates cognitive dysfunction caused by PFOS exposure in mice

To evaluate the effects of ICT on PFOS-induced cognitive dysfunction, mice were orally administered PFOS (50 mg/kg/day) for 28 consecutive d, with concurrent treatment of ICT at corresponding doses (once daily for 28 d), followed by behavioral testing (Figure 1A). Cognitive function was assessed through a series of behavioral tests. The results showed that ICT significantly and dose-dependently increased the discrimination index in PFOS-exposed mice (Figure 1B,C). In the Morris water maze (MWM) test, PFOS exposure markedly prolonged escape latency, reduced platform crossings, and decreased time spent in the target quadrant. These cognitive deficits were significantly ameliorated by treatment with various doses of ICT or donepezil (Figure 1D–G). Importantly, no significant differences in swimming speed were observed across groups (Figure 1H), ruling out potential confounding effects of motor dysfunction on cognitive performance. Consistent with these findings, the Y-maze test revealed that PFOS exposure significantly reduced spontaneous alternation, which was effectively restored by both ICT and donepezil treatment (Figure 1I,J). These results indicate that PFOS exposure induces significant cognitive impairment in mice, while ICT treatment markedly mitigates PFOS-induced cognitive deficits.

### 3.2. ICT attenuates PFOS-induced hippocampal astrocytic activation, Aβ deposition, neuroinflammation and cerebral oxidative stress

**Figure 2.**
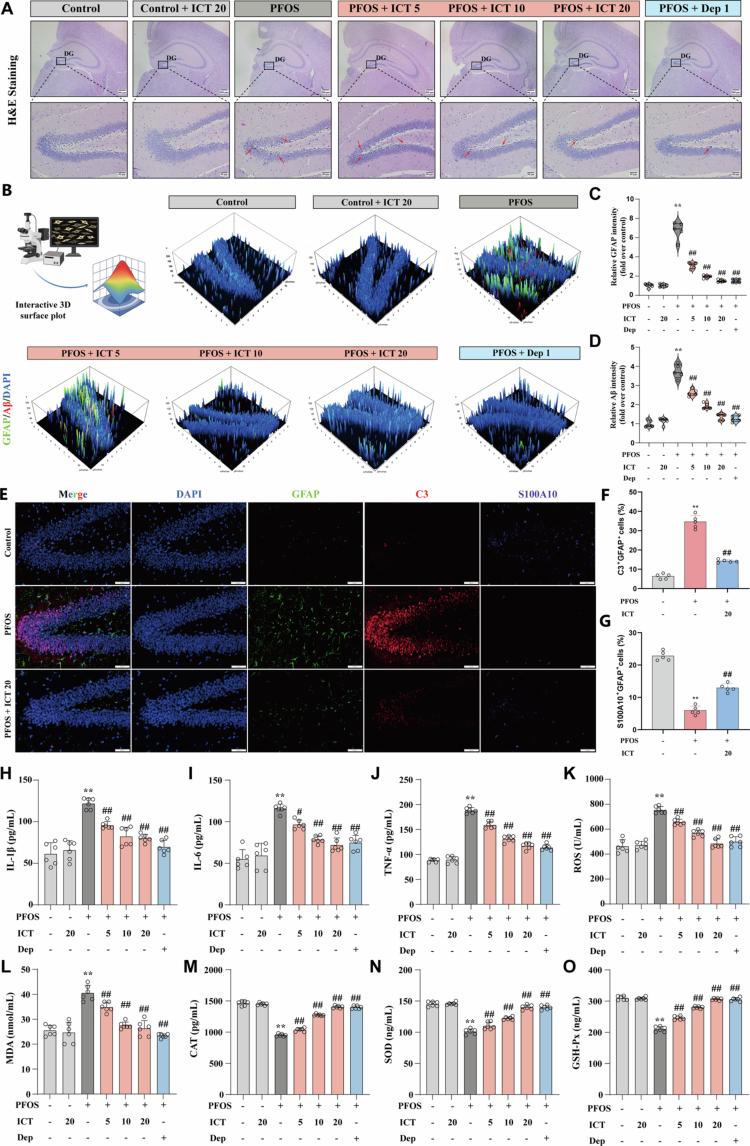
ICT alleviates PFOS-induced activation of hippocampal astrocytes and Aβ pathology, neuroinflammation, and oxidative stress. (A) Representative H&E staining of the hippocampal dentate gyrus (DG) region showing neuronal morphology. Scale bars: 500 μm (upper panel, overview), 50 μm (lower panel, detailed view). (B) Dual immunofluorescence staining of GFAP (green, astrocyte marker) and Aβ (red) in the hippocampal DG region. (C) Quantitative analysis of GFAP fluorescence intensity (*n* = 5). (D) Quantitative analysis of Aβ fluorescence intensity (*n* = 5). (E) Triple-label immunofluorescence staining of GFAP (green), C3 (red) and S100A10 (purple) in the hippocampal DG region. (F) Quantitative analysis of C3^+^ GFAP^+^ cells (%) (*n* = 5). (G) Quantitative analysis of S100A10^+^ GFAP^+^ cells (%) (*n* = 5). (H−J) Hippocampal levels of pro-inflammatory cytokines: (H) IL-1β (*n* = 6), (I) IL-6 (*n* = 6), and (J) TNF-*α* (*n* = 6), measured by ELIZA. (K) Reactive oxygen species (ROS) levels in hippocampal tissues (*n* = 6). (L) Malondialdehyde (MDA) content, a lipid peroxidation marker (*n* = 6). (M−O) Activities of antioxidant enzymes: (M) catalase (CAT, *n* = 6), (N) superoxide dismutase (SOD, *n* = 6), and (O) glutathione peroxidase (GSH-Px, *n* = 6) in hippocampal tissues. The data were presented as the mean ± SEM. ^**^P < 0.01 *vs.* Control group; ^#^*P* < 0.05, ^##^*P* < 0.01 *vs.* PFOS group.

To investigate the neuroprotective effects of ICT against PFOS-induced neurotoxicity, histopathological alterations in mouse brain tissue were first examined using Hematoxylin and eosin (H&E) staining. PFOS exposure induced notable neuronal damage in the hippocampal dentate gyrus (DG), characterized by pyknotic nuclei with intense basophilia and disorganized cytoarchitecture (Figure 2A). Since astrocyte activation and Aβ deposition are closely associated with cognitive decline, we further investigated these pathological features using double immunofluorescence staining. PFOS significantly enhanced both astrocyte activation and Aβ deposition in the hippocampus. ICT treatment markedly reduced the fluorescence intensity of GFAP and Aβ (Figure 2B–D). Furthermore, to better characterize the PFOS-induced astrocyte activation phenotype, we performed triple-label immunofluorescence co-staining for GFAP along with the A1-specific marker C3 and the A2-specific marker S100A10 in the DG. Quantitative analysis revealed a significant increase in GFAP^+^/C3^+^ double staining intensity in the PFOS group compared with the Control group, indicating a clear shift toward the neurotoxic A1 phenotype (Figure 2E,F). In contrast, PFOS exposure significantly reduced GFAP^+^/S100A10^+^ double-staining intensity, suggesting a concomitant suppression of the neuroprotective A2 phenotype (Figure 2E,G). Importantly, co-treatment with ICT effectively mitigated these alterations, significantly lowering the A1-associated signal (GFAP^+^/C3^+^) signal and elevating the A2-associated GFAP^+^/S100A10^+^ signal relative to the PFOS group (Figure 2E,G). These results indicate that PFOS promotes reactive astrogliosis skewed predominantly toward the detrimental A1 polarization, and that ICT confers protection by counteracting this polarization shift, suggesting its potential to interrupt the detrimental cycle between reactive astrogliosis and Aβ pathology, thereby contributing to cognitive improvement. Furthermore, ICT administration notably suppressed the elevated levels of pro-inflammatory cytokines (IL-1β, IL-6, and TNF-*α*) (Figure 2H–J), reduced oxidative stress markers (ROS and MDA) (Figure 2K,L), and restored the activity of key antioxidant enzymes (CAT, SOD, and GSH-Px) (Figure 2M–O). Collectively, these data demonstrate that ICT alleviates PFOS-induced cognitive impairment through multiple pathways, including the attenuation of hippocampal astrocytic activation and Aβ deposition, as well as the reduction of neuroinflammation and oxidative stress. These mechanisms synergistically contribute to the restoration of neuronal homeostasis and cognitive function.

### 3.3. ICT ameliorates PFOS-induced gut microbiota dysbiosis, and suppresses the ammonia-producing bacteria in mice

**Figure 3.**
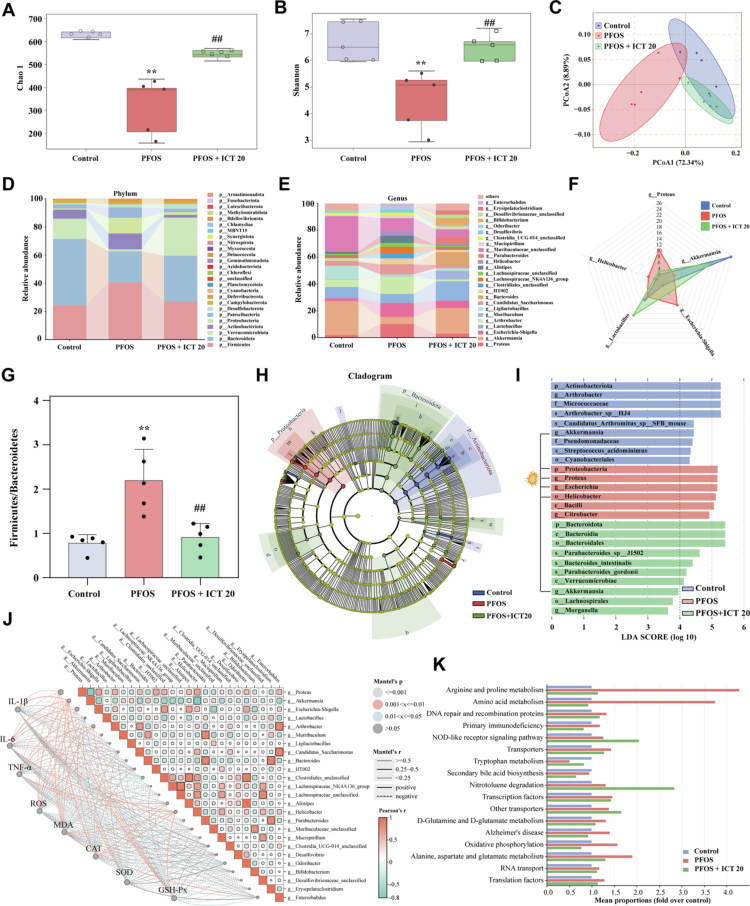
ICT ameliorates PFOS-induced gut microbiota dysbiosis in mice. The impact of ICT on gut microbial composition was assessed through comprehensive 16S rRNA sequencing analysis of fecal samples collected after 28 d of treatment. (A) Chao1 index and (B) Shannon index was used to evaluate microbial *α*-diversity, representing species richness and diversity, respectively. (C) Principal coordinates analysis (PCoA) based on Bray-Curtis distances visualized the *β*-diversity of microbial communities, showing distinct clustering patterns among groups. (D) Relative abundance of bacterial taxa at the phylum level and (E) genus level demonstrate the taxonomic shifts induced by PFOS exposure and ICT intervention. (F) Detailed profiling of the top 5 most abundant bacterial genera provides species-specific information. (G) Firmicutes/Bacteroidetes (F/B) ratio, an important indicator of gut microbiota balance, shows significant alterations across experimental groups. (H) Dendrogram illustrating the hierarchical clustering of microbial communities, with blue representing control, red representing PFOS, and green representing PFOS + ICT 20 mg/kg groups. (I) Linear discriminant analysis Effect Size (LEfSe) analysis identified differentially abundant bacterial taxa among control, PFOS, and PFOS + ICT 20 groups. (J) Correlation analysis between gut microbiota alterations and host physiological parameters, performed using Mantel test with Pearson correlation. (K) Functional profiling of microbial genes predicted through PICRUSt analysis. The data were presented as the mean ± SEM (*n* = 5). ^**^P < 0.01 *vs.* Control group; ^#^*P* < 0.05, ^##^*P* < 0.01 *vs.* PFOS group.

To investigate the effects of ICT on gut microbiota composition in PFOS-exposed mice, we performed 16S rRNA sequencing. Mice with PFOS-induced cognitive dysfunction exhibited a significant reduction in gut microbiota *α*-diversity compared to controls, whereas ICT treatment (20 mg/kg) effectively restored *α*-diversity, as indicated by increased Chao1 and Shannon indices (Figure 3A,B). Principal coordinates analysis (PCoA) revealed clear separation among the experimental groups, with ICT shifting the microbial community structure toward that of the control group pattern (Figure 3C). At the phylum level, PFOS exposure increased the relative abundances of *Firmicutes* and *Proteobacteria*, and decreased those of *Bacteroidetes* and *Verrucomicrobia*. ICT treatment reversed these alterations, restoring microbial composition to near-baseline levels (Figure 3D). At the genus level, PFOS reduced the beneficial bacteria such as *Akkermansia* and *Lactobacillus*, and increased potentially harmful taxa including *Helicobacter*, *Proteus*, and *Escherichia-Shigella*, all of which these alterations were normalized by ICT administration (Figure 3E,F). Notably, ICT significantly decreased the *Firmicutes*/*Bacteroidetes* ratio (Figure 3G). Cladogram and LEfSe analyzes further confirmed substantial alterations in microbial taxa abundance, underscoring the modulatory effect of ICT on gut microbiota in PFOS-exposed mice (Figure 3H,I). Correlation analysis revealed that *Helicobacter*, *Proteus*, and *Escherichia-Shigella* were positively correlated with proinflammatory cytokines and oxidative stress markers, and negatively correlated with antioxidant enzyme levels (Figure 3J). Functional prediction *via* PICRUSt2 indicated that the protective effects of ICT were mediated primarily through ammonia-related metabolic pathways, particularly arginine and proline metabolism, amino acid metabolism, and tryptophan metabolism (Figure 3K). These findings provide mechanistic insights into how ICT-mediated gut microbiota remodeling confers protection against PFOS-induced cognitive impairment.

### 3.4. ICT ameliorates PFOS-induced gut microbiome remodeling and restores ammonia metabolic homeostasis, reducing intestinal-derived ammonia

**Figure 4.**
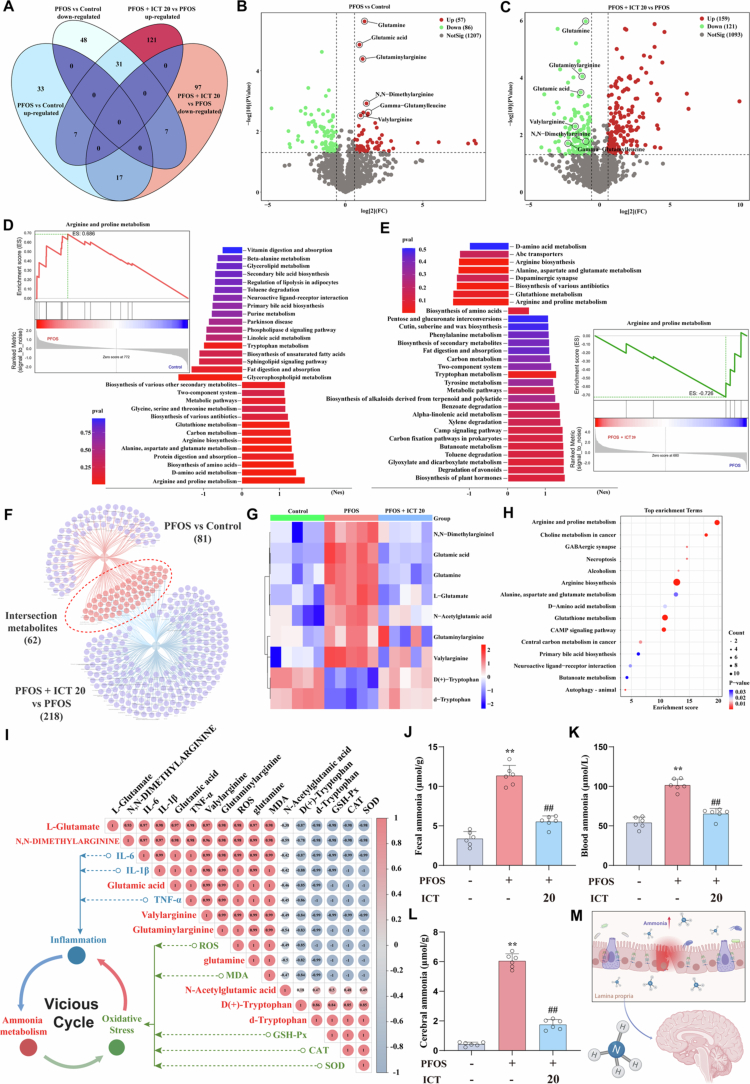
ICT attenuates PFOS-induced dysbiosis and reduces intestinal ammonia production. Comprehensive metabolomic analysis was performed using untargeted metabolomics analysis to characterize fecal metabolites. (A) Venn diagram illustrating the number of significantly upregulated and downregulated metabolites across different comparison groups. (B−C) Volcano plots displaying differential metabolites in (B) PFOS *versus* control group and (C) PFOS + ICT (20 mg/kg) *versus* PFOS group. (D−E) Gene Set Enrichment Analysis (GSEA) showing significantly altered metabolic pathways in (D) PFOS *versus* control and (E) PFOS + ICT *versus* PFOS comparisons. (F) Venn diagram of overlapping significantly altered metabolites among different experimental groups. (G) Heatmap visualization of key metabolites involved in ammonia metabolism pathways, demonstrating distinct clustering patterns among treatment groups. (H) KEGG pathway enrichment analysis of significantly altered metabolites. (I) Correlation heatmap analyzing relationships between differential metabolites and inflammation and oxidative stress using Spearman correlation coefficients. (J−L) Quantitative measurements of ammonia levels in (J) fecal samples (*n* = 6), (K) blood serum (*n* = 6), and (L) cerebral tissues (*n* = 6) using commercial ELIZA kits. (M) Schematic diagram summarizing the potential mechanism by which ICT modulates gut microbiota and metabolic pathways to reduce ammonia production and alleviate PFOS-induced toxicity. The data were presented as the mean ± SEM. ^**^P < 0.01 *vs.* Control group; ^#^*P* < 0.05, ^##^*P* < 0.01 *vs.* PFOS group.

Given the modulatory effects of ICT on gut microbiota, we conducted untargeted metabolomics analysis to identify associated microbial metabolites. Compared with controls, PFOS exposure induced significant perturbations in the metabolite profiles, which were effectively reversed by ICT treatment (Figure 4A). Using differential analysis thresholds of fold change >1.5, Q-value < 0.05, and VIP > 1, we identified 57 upregulated and 86 downregulated metabolites in the PFOS group compared to controls (Figure 4B). Notably, the PFOS + ICT (20 mg/kg) group exhibited 159 increased and 121 decreased metabolites compared to PFOS group. Among these, six key metabolites related to ammonia metabolism, which were elevated by PFOS, were reduced following ICT intervention (Figure 4C). Gene set enrichment analysis (GSEA) revealed that PFOS-induced cognitive impairment was associated with upregulation of arginine and proline metabolism and amino acid biosynthesis, along with downregulated tryptophan metabolism, all of which are pathways implicated in ammonia metabolism. ICT treatment effectively reversed these metabolic pathway alterations (Figure 4D,E). Furthermore, 62 differentially abundant metabolites were common across comparisons (Figure 4F). Heatmap visualization demonstrated pronounced intergroup differences in ammonia-related metabolites (valylarginine, glutaminyl arginine, glutamine), which were elevated in the PFOS group and normalized by ICT (Figure 4G). KEGG pathway analysis confirmed arginine and proline metabolism as the most significantly enriched pathway, consistent with 16S rRNA findings (Figure 4H). Correlation analysis showed positive associations between these metabolites and proinflammatory cytokines and oxidative stress markers, and negative correlations with antioxidant enzymes, suggesting their potential role in ICT-mediated cognitive improvement (Figure 4I). We further quantified ammonia levels in feces, serum and brain tissue, and observed that ICT markedly reduced ammonia concentrations in all compartments (Figure 4J–L). Additional investigations indicated that elevated cerebral ammonia was accompanied by suppressed astrocytic GS activity, reduced Gln production, and decreased expression of carbamoyl phosphate synthetase 1 (CPS-1) (Figure S1A–C), indicating compromised cerebral ammonia detoxification. ICT treatment effectively restored GS activity, Gln levels, and CPS-1-related pathways. These comprehensive results demonstrate that ICT confers neuroprotection by modulating gut microbiota composition, reducing intestinal ammonia, and attenuating its accumulation in the brain, thereby alleviating cerebral oxidative stress and neuroinflammation (Figure 4M). This coordinated multi-omics evidence positions ammonia metabolism as a central mechanistic link between gut microbial remodeling and cognitive recovery.

### 3.5. ICT modulates gut microbiota, attenuates inflammatory responses, and preserves intestinal barrier integrity against PFOS-induced injury

**Figure 5.**
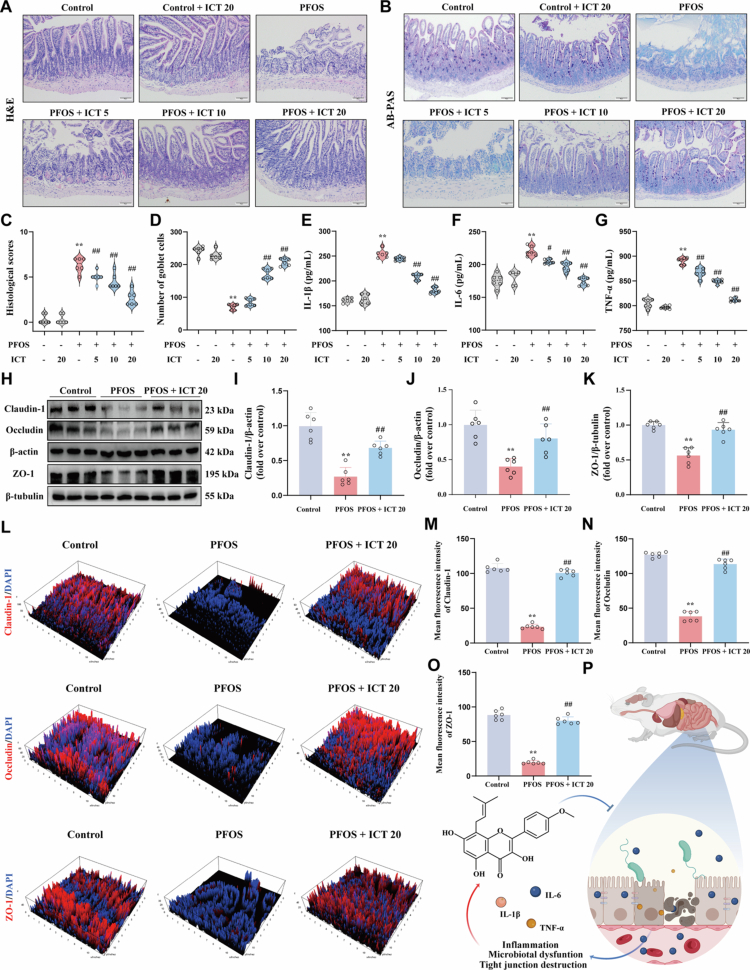
ICT modulates the gut microbiota, suppresses inflammation, and protects the intestinal barrier from PFOS-induced disruption. (A) Representative H&E staining images of ileum tissues showing histological alterations, with scale bar = 50 μm. (B) Alcian blue-periodic acid-Schiff (AB-PAS) staining images illustrating goblet cell distribution, with scale bar = 50 μm. (C) Quantitative histological scores based on H&E staining evaluation. (D) Quantification of goblet cell numbers derived from AB-PAS staining. (E−G) Levels of pro-inflammatory cytokines in ileum tissues measured by ELIZA: (E) IL-1β, (F) IL-6, and (G) TNF-*α*. (H) Western blot analysis of tight junction proteins (Claudin-1, Occludin, and ZO-1) in ileum tissues. (I−K) Densitometric quantification of protein expression: (I) Claudin-1, (J) Occludin, and (K) ZO-1, normalized to *β*-actin. (L) Immunofluorescence (IF) imaging of tight junction structures using antibodies against Claudin-1, Occludin, and ZO-1. (M−O) Mean fluorescence intensity quantification from IF images: (M) Claudin-1, (N) Occludin, and (O) ZO-1. (P) Schematic illustration summarizing the proposed mechanism by which ICT modulates gut microbiota to suppress inflammation and restore intestinal barrier integrity after PFOS exposure. The data were presented as the mean ± SEM (*n* = 6). ^**^P < 0.01 *vs.* Control group; ^#^*P* < 0.05, ^##^*P* < 0.01 *vs.* PFOS group.

To investigate the role of the microbiota-gut-brain axis in ICT-mediated protection, we examined the effects of ICT on PFOS-induced intestinal damage. Histopathological analysis revealed that PFOS exposure caused severe intestinal injury, characterized by villus atrophy, disruption of ileal structural, and depletion of goblet cells (Figure 5A–D), which was accompanied by significant inflammatory responses including elevated levels of IL-1β, IL-6, and TNF-*α* (Figure 5E–G). ICT treatment dose-dependently restored goblet cell numbers and normalized cytokine levels, demonstrating a protective effect on intestinal integrity. Furthermore, ICT (20 mg/kg) significantly upregulated the key expression of tight junction proteins (Claudin-1, Occludin, and ZO-1) compared to PFOS group (Figure 5H–K). Immunofluorescence staining corroborated these findings, revealing PFOS exposure markedly reduced the expression of these tight junction proteins, while ICT treatment effectively restored their levels (Figure 5L–O). These findings demonstrate that ICT enhances intestinal barrier function by modulating gut microbiota homeostasis, thereby suppressing inflammatory responses and ameliorating PFOS-induced intestinal epithelial damage (Figure 5P). Based on these data, ICT effectively counteracts PFOS-induced intestinal damage by restoring gut microbial balance, strengthening the epithelial barrier, and dampening local inflammatory signaling.

### 3.6. ICT ameliorates intestinal barrier dysfunction and inhibits PFOS-induced cognitive impairment through the gut-brain axis

**Figure 6.**
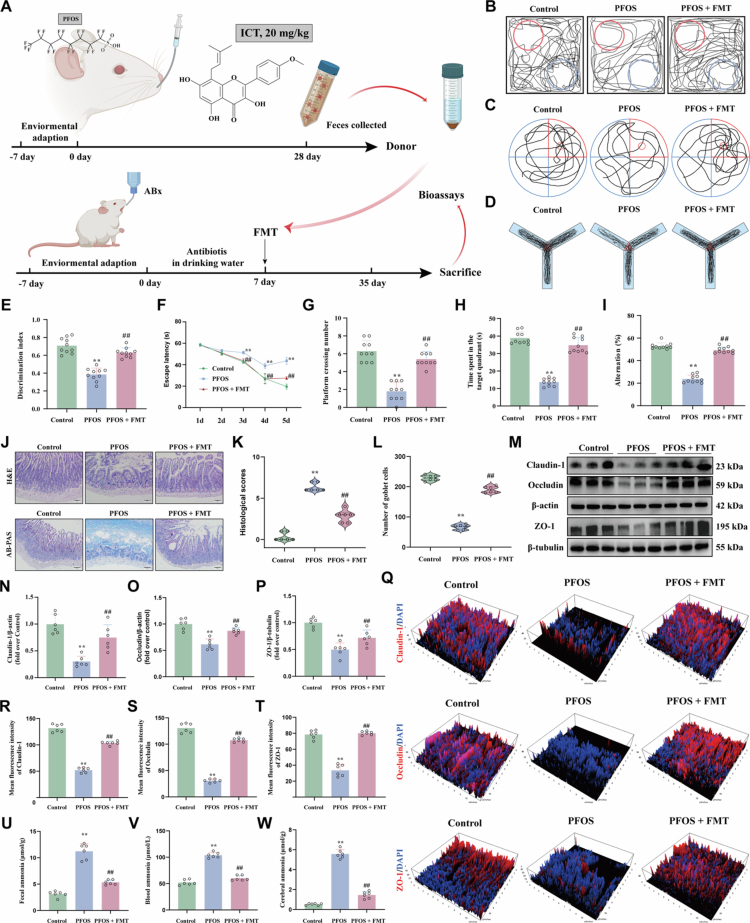
The neuroprotective effect of ICT against PFOS-induced cognitive impairment is mediated through modulation of the gut-brain axis. (A) Schematic diagram of the experimental design for animal experiment 2, illustrating the FMT procedure from donor mice to antibiotic-pretreated recipient mice. (B) Representative search trajectories of mice in the NOR test during the testing phase. (C) Representative swimming trajectories of mice in the MWM probe trial. (D) Representative movement trajectories of mice in the Y-maze test. (E) Discrimination index in the NOR test (*n* = 10). (F) Escape latency during the 5-day acquisition phase of the MWM test (*n* = 10). (G) Number of platform crossings during the MWM probe trial (*n* = 10). (H) Time spent in the target quadrant during the MWM probe trial (*n* = 10). (I) Spontaneous alternation rate in the Y-maze test (*n* = 10). (J) Representative histological images of ileum tissues: H&E staining (upper panel) and AB-PAS staining (lower panel) with scale bar = 50 μm. (K) Quantitative histological scores based on H&E staining evaluation (*n* = 6). (L) Goblet cell counts per villus derived from AB-PAS staining (*n* = 6). (M) Representative Western blot images of tight junction proteins (Claudin-1, Occludin, and ZO-1) in ileum tissues. (N−P) Quantification of tight junction protein expression: (N) Claudin-1 (*n* = 6), (O) Occludin (*n* = 6), and (P) ZO-1 (*n* = 6). (Q) Representative immunofluorescence images of tight junction structures stained for Claudin-1, Occludin, and ZO-1. (R−T) Mean fluorescence intensity quantification: (R) Claudin-1 (*n* = 6), (S) Occludin (*n* = 6), and (T) ZO-1 (*n* = 6). (U−W) Ammonia levels measured by ELIZA in: (U) fecal samples (*n* = 6), (V) blood serum (*n* = 6), and (W) cerebral tissues (*n* = 6). The data were presented as the mean ± SEM. ^**^P < 0.01 *vs.* Control group; ^#^*P* < 0.05, ^##^*P* < 0.01 *vs.* PFOS group.

To further assess whether ICT-modulated intestinal microbiota is sufficient to confer protection against cognitive dysfunction, we established a pseudo-germ-free mouse model by pretreatment with a broad-spectrum antibiotic cocktail (ABX) for 7 d prior to PFOS exposure. FMT was then performed using donor microbiota from ICT-treated donors significantly alleviated PFOS-induced cognitive deficits (Figure 6A). Behavioral analyzes demonstrated that FMT derived from ICT-treated donors significantly alleviated PFOS-induced cognitive impairment. Specifically, FMT increased the discrimination index in the novel object recognition test, shortened escape latency in the MWM, increased platform crossings and time spent in target quadrant, and restored spontaneous alternation behavior in the Y-maze test (Figure 6B–I). Histological evaluation revealed that FMT recipients exhibited markedly improved histological morphology, including reduced histopathological scores and increased goblet cell numbers compared to PFOS-exposed mice (Figure 6J–L). Western blot analysis further revealed that FMT significantly enhanced the expression of tight junction proteins (Claudin-1, Occludin, and ZO-1) relative to the PFOS group (Figure 6M–P), a finding corroborated by immunofluorescence staining (Figure 6Q–T). Notably, FMT significantly reduced ammonia levels in feces, serum, and brain tissue (Figure 6U–W). Consistently, microbiota analysis showed that FMT restored microbial homeostasis, similarly to direct ICT treatment, by reducing ammonia-producing pathobionts (e.g., Helicobacter, Proteus, and Escherichia–Shigella) and enriching beneficial genera such as Akkermansia and Lactobacillus (Figure S2A–D). Additionally, FMT modulated key ammonia-metabolizing enzymes, influencing the activities of GS, and CPS-1 and the level of Gln (Figure S2E–G). These results indicate that ICT-mediated remodeling of the gut microbiota attenuates gut-derived ammonia dysmetabolism and intestinal barrier impairment, thereby regulating the gut-brain axis and ultimately mitigating PFOS-induced cognitive decline.

### 3.7. Intestinal ammonia metabolic homeostasis is pivotal for ICT-mediated protection against PFOS-induced cognitive dysfunction

**Figure 7.**
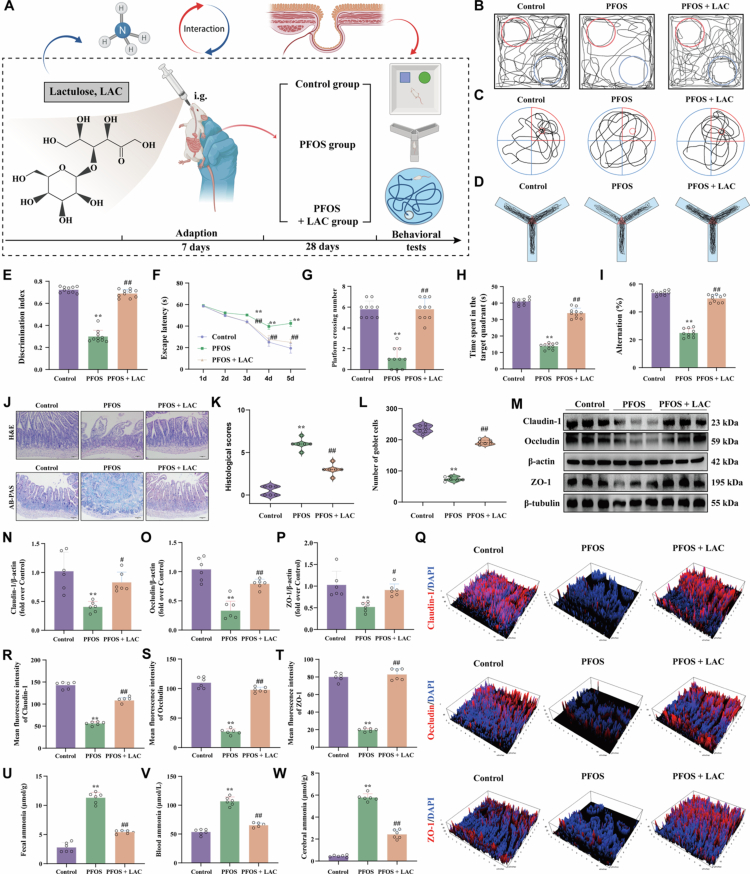
Intestinal ammonia metabolic homeostasis is essential for ICT-mediated protection against PFOS-induced cognitive dysfunction. (A) Experimental design schematic illustrating the lactulose intervention study, where mice received PFOS exposure with or without lactulose for 28 d. (B) Representative search trajectories of mice in the NOR test during the testing phase. (C) Representative swimming trajectories of mice in the MWM probe trial. (D) Representative movement trajectories of mice in the Y-maze test. (E) Discrimination index in the NOR test (*n* = 10). (F) Escape latency during the 5-day acquisition phase of the MWM test (*n* = 10). (G) Number of platform crossings during the MWM probe trial (*n* = 10). (H) Time spent in the target quadrant during the MWM probe trial (*n* = 10). (I) Spontaneous alternation rate in the Y-maze test (*n* = 10). (J) Representative histological images of ileum tissues: H&E staining (upper panel) and AB-PAS staining (lower panel) with scale bar = 50 μm. (K) Quantitative histological scores based on H&E staining evaluation (*n* = 6). (L) Goblet cell counts per villus derived from AB-PAS staining (*n* = 6). (M) Representative Western blot images of tight junction proteins (Claudin-1, Occludin, and ZO-1) in ileum tissues. (N−P) Quantification of tight junction protein expression: (N) Claudin-1 (*n* = 6), (O) Occludin (*n* = 6), and (P) ZO-1 (*n* = 6). (Q) Representative immunofluorescence images of tight junction structures stained for Claudin-1, Occludin, and ZO-1. (R-T) Mean fluorescence intensity quantification: (R) Claudin-1 (*n* = 6), (S) Occludin (*n* = 6), and (T) ZO-1 (*n* = 6). (U−W) Ammonia levels measured by ELIZA in: (U) fecal samples (*n* = 6), (V) blood serum (*n* = 6), and (W) cerebral tissues (*n* = 6). The data were presented as the mean ± SEM. ^**^P < 0.01 *vs.* Control group; ^#^*P* < 0.05, ^##^*P* < 0.01 *vs.* PFOS group.

Our study demonstrates that PFOS exposure disrupts gut microbiota homeostasis, elevates intestinal-derived ammonia levels, and consequently exacerbates cerebral ammonia toxicity. Accumulating evidence indicates that excessive systemic ammonia accumulation induces significant neurotoxicity, disrupts cerebral ammonia metabolism, and facilitates the accumulation of toxic metabolites.^37-41^ Therefore, reducing intestinal ammonia levels may represent a critical strategy for mitigating ammonia-associated cognitive impairment. To test this hypothesis, lactulose (LAC), which is a known ammonia-lowering agent, was administered orally to attenuate intestinal-derived ammonia generation and potentially alleviate neurotoxicity (Figure 7A). LAC significantly ameliorated PFOS-induced cognitive deficits, as evidenced by increased discrimination index in novel object recognition tests, shortened escape latency in the MWM, increased platform crossings and time spent in the target quadrant, and improved spontaneous alternation in the Y-maze (Figure 7B–I). Furthermore, LAC reduced ileal histopathological scores and increasing goblet cell numbers (Figure 7J–L), Western blot analysis showed that LAC markedly upregulated the levels of key tight junction proteins (Claudin-1, Occludin, and ZO-1) compared to the PFOS group (Figure 7M–P), a finding corroborated by immunofluorescence staining (Figure 7Q–T). Importantly, LAC effectively reduced ammonia levels in feces, serum and brain tissue (Figure 7U–W). Notably, although LAC effectively reduced systemic ammonia and improved cognitive function, it did not reverse PFOS-induced gut microbial dysbiosis (Figure S3A–C), suggesting that its actions may involve direct chemical modulation or non-microbiota-dependent pathways rather than structural remodeling of the gut microbiota. Conversely, similar to ICT, LAC restored the activity of key cerebral ammonia metabolism, including GS activity and CPS-1, and normalized Gln levels (Figure S3D–F). Collectively, these results demonstrate that reducing intestinal ammonia alleviates PFOS-induced cognitive dysfunction. Although LAC and ICT differentially affect gut microbial composition, both interventions improve ammonia metabolic homeostasis and confer neuroprotection. These findings further indicate that the protective effects of ICT are mediated, at least in part, through gut microbiota-dependent regulation of ammonia metabolism, resulting in decreased intestinal ammonia production and attenuated cerebral ammonia toxicity.

## 4. Discussion

**Figure 8.**
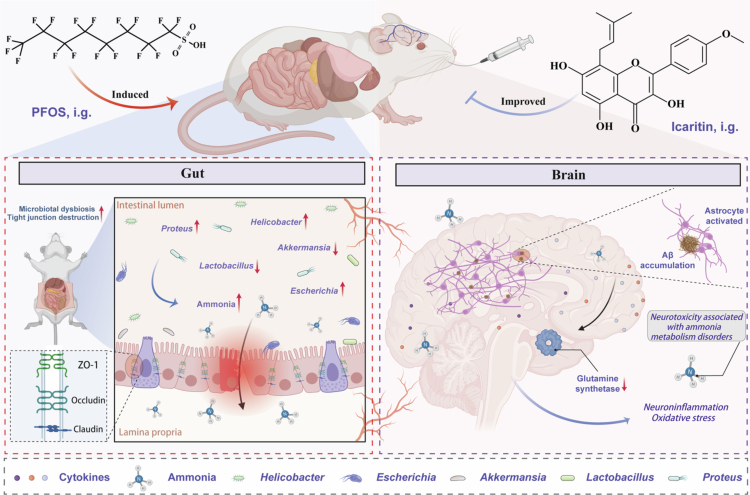
Schematic illustrating the proposed mechanism by which ICT alleviates PFOS-induced cognitive dysfunction through gut microbiota remodeling and ammonia metabolic regulation.​ PFOS induces gut microbiota dysbiosis, increasing ammonia production. Elevated ammonia enters circulation, impairing astrocyte function, triggering neuroinflammation/oxidative stress, and causing cognitive deficits. ICT remodels gut microbiota, normalizes ammonia metabolism, and alleviates these pathological changes, demonstrating gut-brain axis mediation.

This study provides evidence suggesting that ICT, a bioactive flavonoid from Epimedium, ameliorates PFOS-induced cognitive dysfunction by modulating the gut-brain axis through ammonia metabolism. Our findings support a mechanism whereby PFOS disrupt gut-brain communication *via* microbial dysbiosis and ammonia dysregulation, and demonstrate the potential of natural compounds in counteracting environmental neurotoxicity (Figure 8).

A pivotal finding of this work is the identification of ammonia as a key mechanistic link connecting gut microbiota disruption to cognitive impairment following PFOS exposure. It is noteworthy that PFOS exposure specifically enriched bacterial genera possessing potent urease and amino acid deaminase activities, such as *Proteus* and *Escherichia-Shigella*. These bacteria are recognized as major producers of ammonia in the gut,^42^^,^^43^ and their proliferation directly explains the systemic hyperammonemia observed in our PFOS model. This dysbiosis led to elevated systemic and cerebral ammonia levels, along with marked perturbations in arginine and proline metabolism pathways. Collectively our data outline a potential mechanistic framework for ammonia-mediated neurotoxicity. Elevated brain ammonia likely impairs astrocytes directly by inhibiting glutamine synthetase (GS) activity. This inhibition not only compromises cerebral ammonia clearance but also disrupts the glutamine cycle. As a consequence, astrocytes with impaired GS are prone to adopting a pro-inflammatory phenotype, releasing cytokines such as IL-1β and TNF-*α*, and thereby exacerbating neuroinflammation. Concurrently, astrocyte dysfunction and the persistent inflammatory environment jointly impair Aβ clearance while potentially promote Aβ production, ultimately leading to Aβ deposition. Thus, through the central hub of astrocyte dysfunction, ammonia likely triggers both neuroinflammation and Aβ pathology, which represent two key pathogenic pathways.

Intervention with ICT effectively restored microbial ecology and reestablished ammonia homeostasis, which in turn mitigated these metabolic disturbances. Importantly, when fecal microbiota transplantation (FMT) was performed using donors treated with ICT and recipients exposed to PFOS, the recipients exhibited comparable cognitive benefits and reduced ammonia burden. This outcome provides direct evidence for the causal role of gut microbiota in this pathway. To clarify whether ICT's effects are mediated directly or through the gut microbiota, we performed an FMT experiment. Crucially, this design is crucial as it effectively distinguishes between direct neuronal effects and indirect effects mediated by microbiota. The results showed that transplanting microbiota from ICT-treated donors into recipients that did not themselves receive ICT successfully reproduced key protective effects. These included lower ammonia levels, improved gut barrier function, and alleviated cognitive impairment. This finding strongly demonstrates that, in our model, the neuroprotective effects of ICT are achieved primarily through modulating gut microbiota rather than through direct actions on neural cells. Although a direct central effect of ICT cannot be completely excluded, the FMT experiment indicates that the microbiota-mediated pathway is a major and essential mechanism for its efficacy in vivo.

Further mechanistic investigations revealed that lactulose (LAC), which directly reduces intestinal ammonia absorption without altering the microbial community structure, also lowered circulating ammonia and alleviated cognitive deficits. This finding robustly demonstrates that elevated ammonia is not only necessary but sufficient to mediate PFOS-induced neurotoxicity. Another consideration is the high PFOS dose used here for mechanistic exploration. While this dose is justified for reliably establishing a cognitive impairment model and is supported by precedent in previous studies,^44^ it may induce broad systemic toxicity. However, the lactulose intervention experiment showed that specifically reducing ammonia levels without modifying the microbial structure improved cognitive function. This indicates that the gut microbiota-ammonia metabolism axis constitutes a relatively independent and targetable pathogenic pathway, even within a complex systemic toxicity background. Nevertheless, validating the universality of this pathway at lower, environmentally relevant exposure levels is crucial for assessing its public health significance and represents an important direction for future research.

In contrast to LAC, ICT operates through a microbiota-dependent strategy that concurrently rectifies dysbiosis and augments the host’s innate detoxification mechanisms. Specifically, ICT strengthens intestinal barrier function, reduces neuroinflammatory signaling, and restores astrocytic GS activity, thereby supporting both systemic and cerebral ammonia clearance. In addition, although gut microbiota and ammonia metabolism primarily occur in the colon, our focus on the ileum is justified because the distal ileum serves as a critical barrier and regulatory gatekeeper, controlling the passage of substances from the intestinal lumen into the systemic circulation. The disruption of its barrier function represents a key step in the translocation of colonic-derived metabolites such as ammonia into the bloodstream, thereby activating the gut-brain axis. While fecal metabolomics and systemic ammonia levels were used to monitor the functional output of the colon, the histological and molecular findings in the ileum provide direct evidence of the consequences, specifically gut barrier failure and systemic inflammation. Future studies that directly examine the colonic barrier and its local microenvironment will yield a more comprehensive understanding of the full scope of PFOS-induced enterotoxicity.

Collectively this study systematically elucidates the protective mechanisms of ICT. Several limitations, however, warrant further investigation. First, although antibiotic treatment confirmed the essential role of gut microbiota, the specific contributions of key bacterial strains require validation through targeted approaches such as mono-colonization experiments. Second, the spatiotemporal dynamics of critical metabolic enzymes across different brain regions remains unclear. Third, the use of a relatively high PFOS dose, though necessary to establish a robust model for mechanistic exploration, may not fully recapitulate the effects of chronic, low-level environmental exposure. Consequently, the quantitative contribution of the gut microbiota-ammonia axis to neurotoxicity at environmentally relevant PFOS levels requires further study. Finally, human data are needed to validate these preclinical findings. Although direct clinical intervention studies on PFOS exposure pose ethical and practical challenges, the translational potential of this work could be explored through alternative approaches. Future research may include epidemiological studies correlating PFOS exposure with gut microbial markers and blood ammonia in human cohorts, or experimental validation using human-derived *in vitro* models (e.g., gut organoids, blood-brain barrier models) to validate the implicated signaling pathways.

In conclusion, this study delineates a previously unrecognized pathway through which PFOS impairs cognitive function via gut microbiota-derived ammonia and establishes ICT as a therapeutic agent capable of disrupting this pathway. Our work underscores the importance of a holistic approach to understanding environmental neurotoxicity. Future efforts should focus on clinical translation and on exploring synergistic neuroprotective strategies.

## Supplementary Material

Fig s2.docx Fig s2.docx

Clean Supplemental Material.docx Clean Supplemental Material.docx

## Data Availability

The data that support the findings of this study are available on figshare at https://doi.org/10.6084/m9.figshare.30466367.v2.
