# Supplementary figures and images for: Gut microbial ammonia as a mediator of PFOS neurotoxicity and its remediation by the flavonoid Icaritin
Source: Gut Microbes. 2026 Feb 2;18(1):2620125. doi: 10.1080/19490976.2026.2620125 (PMC12885405; doi:10.1080/19490976.2026.2620125)

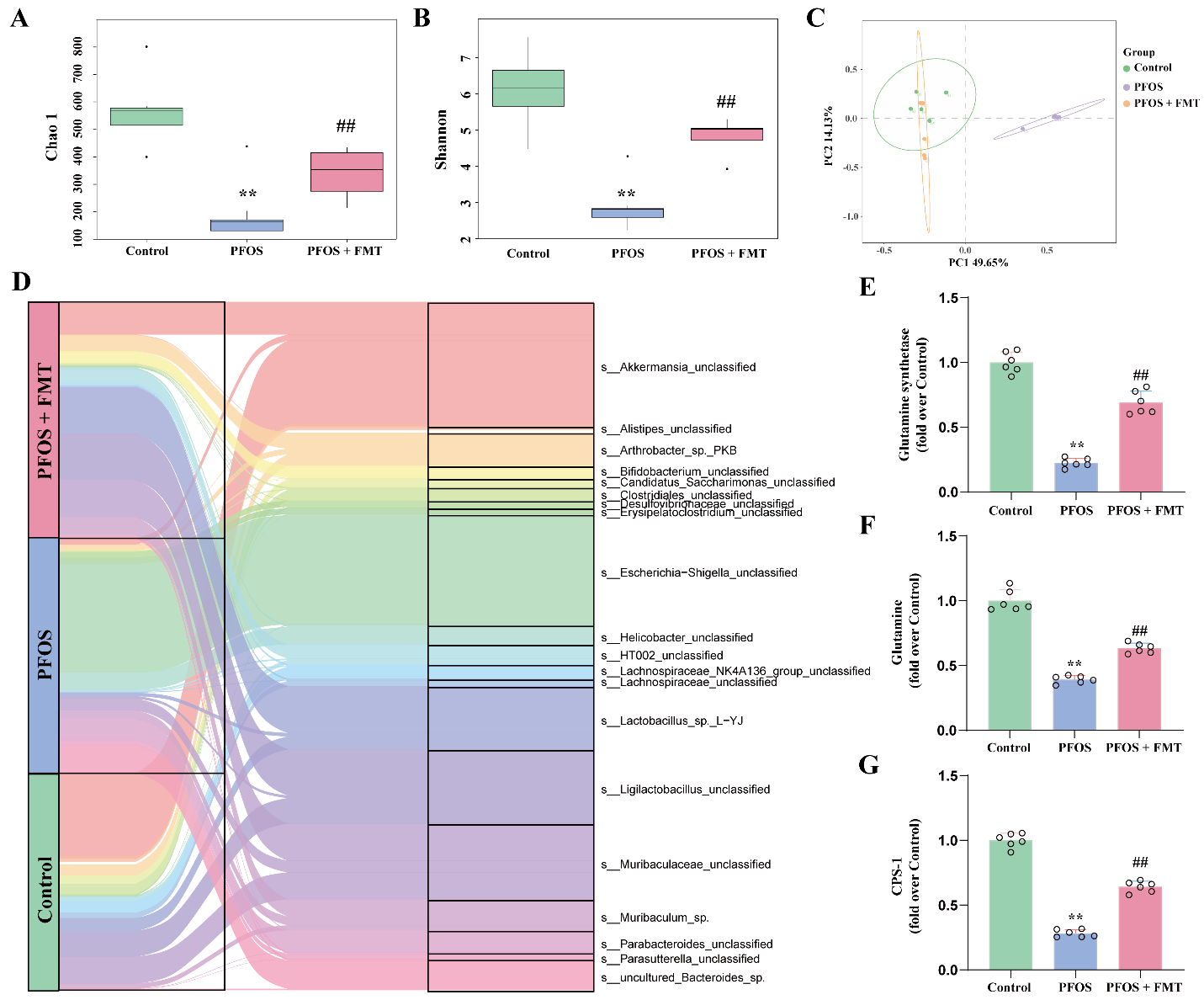

Supplement: Fig s2.docx [file KGMI_A_2620125_SM4077.docx]
