# Supplementary material for: Gut microbial ammonia as a mediator of PFOS neurotoxicity and its remediation by the flavonoid Icaritin
Source: Gut Microbes. 2026 Feb 2;18(1):2620125. doi: 10.1080/19490976.2026.2620125 (PMC12885405; doi:10.1080/19490976.2026.2620125)
Supplement: Clean Supplemental Material.docx [file KGMI_A_2620125_SM4076.docx]

Supplementary Materials for

**Gut microbial ammonia as a mediator of PFOS neurotoxicity and its remediation by the flavonoid Icaritin**

Yang Yi^1,2,3†^, Wenfang Zhang^1,2†^, Yu Wei^1,2†^, Wang Ran^1,2^, Dongjing Liu^1,2^, Weikun Deng^1,2^, Songyuan Duan^1,2^, Jiyong Yao^1,2^, Lianhang Wang^1,2^, Yuandong Zhang^1,2^, Jianmei Gao^1,2^, Qihai Gong^1,2,3*^

^1^Key Laboratory of Basic Pharmacology of Ministry of Education and Joint International Research Laboratory of Ethnomedicine of Ministry of Education, Zunyi Medical University, Zunyi, China

^2^Department of Pharmacology, Key Laboratory of Basic Pharmacology of Guizhou Province and School of Pharmacy, Zunyi Medical University, Zunyi, Guizhou, China

^3^ Department of Pharmacology, Guizhou Medical University, Guiyang, China.

^†^These authors contributed equally to this work.

^*^Corresponding author: Prof. Qihai Gong, Ph.D.

6 Xuefu West Road, Zunyi City, Guizhou Province, 563000, P. R. China

Tel: +86-851-286-423-03

Fax: +86-851-286-423-03

E-mail: gqh@zmu.edu.cn

**Contents**

**1. Supplementary Materials and Methods**

**2. Figure S1.** ICT ameliorates brain ammonia metabolism by enhancing the activity of key detoxification enzymes.

**3. Figure S2.** FMT from ICT-treated donors partially restores PFOS-induced dysbiosis and ameliorates brain ammonia metabolism by enhancing the activity of key detoxification enzymes.​

**4. Figure S3.** LAC alleviates PFOS-induced impairments in ammonia metabolism without altering gut microbiota structure.

**1. Supplementary Materials and Methods**

***Behavioral Tests:*** Novel object recognition (NOR): The NOR test was conducted in a custom-built open-field arena (50 × 50 × 50 cm) according to an established.^[1]^.After a habituation period to the arena, each mouse was permitted to freely explore two identical objects for 10 min. Following an intertrial interval of 10 min, during which the animal was temporarily removed from the arena, the objects were carefully cleaned with ethanol to eliminate residual olfactory cues. One of the familiar objects (F) was then replaced with a novel object (N). Exploratory behavior was defined as the mouse directing its nose toward the object or making physical contact with it using the forepaws. The recognition index, expressed as a percentage, was calculated using the formula: Recognition index = N/ (N + F).

Morris water maze (MWM): Spatial learning and memory were assessed using the MWM test as previously outlined.^[1]^ The apparatus consisted of a circular pool measuring 120 cm in diameter and 50 cm in height, filled with water maintained at 23 ± 1 ℃. The pool was conceptually divided into four quadrants, and a hidden platform (10 cm in diameter) was positioned 1 cm below the water surface in one quadrant. During the 5-day acquisition phase, mice were trained to locate the submerged platform. On day 6, a probe trial was carried out with the platform removed to evaluate spatial memory retention. Several parameters were automatically recorded and analyzed using a video tracking system, including escape latency, time spent in the target quadrant, number of platform crossings, and swimming speed.

Y maze: The Y-maze test was conducted using a maze with three arms of identical dimensions (30 × 10 × 20 cm) in accordance with a previously described protocol.^[1]^ Each mouse was initially placed in one arm and allowed to explore the maze freely for 8 minutes. Following a short interval, the mice was reintroduced to the same starting arm and given 5 minutes to move freely among all three arms. All arm entries were recorded throughout the session. Spontaneous alternation behavior, which reflects working memory performance, was quantified according to the following the formula: percentage spontaneous alternation = [number of alternations / (total arm entries − 2)] × 100.

***Histopathological Assessment:*** Brain and ileum tissues were fixed in 10% formalin for 48 h, then subjected to dehydration (TP1020, Leica) and embedded in paraffin. Sections were cut at a thickness of 4 μm using a microtome (BG-1150, ROM-2245, Leica). Hematoxylin and eosin (H&E) staining was performed with an automated stainer (ST5010, Leica Autostainer XL). Histopathological scoring of ileal injury was conducted based on established criteria that give priority to evaluating two primary aspects, including the severity of inflammation and the integrity of crypt architecture.^[2]^ Three independent pathologists, who were blinded to the experimental groupings, evaluated all sections. Final histopathology scores represent the average of all evaluations.

***AB-PAS Staining:*** Paraffin-embedded ileum tissue sections were deparaffinized and rehydrated prior to staining. AB-PAS staining was performed following the instructions provided with the commercial kit. Briefly, sections were incubated in Alcian blue solution for 10 min and subsequently rinsed three times with distilled water. They were then treated with an oxidizing agent for 5 min and stained with Schiff’s reagent for an additional 10 min. Cell nuclei were counterstained with hematoxylin for 1 min, followed by bluing in Scott’s solution for 3 min. Finally, sections were dehydrated, cleared, and mounted with a transparent medium for microscopic observation (BX43, Olympus, Tokyo, Japan). Goblet cells were quantified using ImageJ.js (v0.5.7; https://ij.imjoy.io).

***Immunofluorescent (IF) Staining:*** IF staining was performed to assess the of astrocyte activation, Aβ distribution, and junction protein expression. Deparaffinized tissue sections were incubated overnight at 4 ℃ with the primary antibodies directed against GFAP (1:100), Aβ_1-42_ (1:100), C3 (1:100), S100A10 (1:100), Claudin-1 (1:100), Occludin (1:100), and ZO-1 (1:100). After washing, the sections were incubated with a fluorescent secondary antibody for 4 h at room temperature. Nuclei were counterstained using an antifade mounting medium containing DAPI. Images were captured with a confocal laser scanning microscope (STELLARIS 5, Leica). Quantitative analysis of fluorescence intensity and 3D surface reconstruction were performed using ImageJ.js (v0.5.7; https://ij.imjoy.io).

***Enzyme-Linked Immunosorbent Assay (ELISA)****:* Tissue samples from the brain and ileum were homogenized in 0.1 M phosphate-buffered saline (PBS, pH 7.4) and centrifuged at 3000 × g for 20 min at 4 ℃. All assays were performed following the manufacturer’s protocols. Inflammatory cytokines levels (IL-1β, IL-6, and TNF-α) in the brain and ileum were quantified using commercial ELISA kits. Additionally, levels of ROS, MDA, CAT, SOD, and GSH-Px in the brain were measured using ELISA kits from the same supplier. Ammonia concentrations in the feces, serum, and brain, as well as the enzymatic activities of GS, and CPS-1 in the brain, were quantified using specific commercial ELISA kits.

***16S rRNA Sequencing:*** To evaluate the influence of ICT on gut microbiota composition in mice with cognitive dysfunction induced by PFOS, fresh fecal samples were collected from the Control group, PFOS, PFOS + ICT (20 mg/kg), PFOS + FMT and PFOS + LAC. Total microbial DNA was extracted using the cetyltrimethylammonium bromide approach. The quality, purity, and integrity of the DNA were verified prior to library preparation. Sequencing was carried out by LC-Bio Co., Ltd. on the Illumina NovaSeq 6000 system. Microbial community and diversity analyses were carried out using the OmicStudio platform (https://www.omicstudio.cn/tool).

***Untargeted Metabolomics:*** Sequencing was carried out by LC-Bio Co., Ltd. Briefly, Fecal samples were collected and kept on ice during processing. Metabolite extraction was carried out using 80% methanol. The extracts were then analyzed by liquid chromatography-mass spectrometry (LC-MS) on an UltiMate 3000 UPLC system equipped with an ACQUITY UPLC T3 column (100 mm × 2.1 mm, 1.8 μm; Waters) maintained at 40 ℃. The mobile phase included solvent A (5 mM ammonium acetate with 5 mM acetic acid) and solvent B (acetonitrile), with a flow rate of 0.3 mL/min. The gradient program was set as follows: 0–0.8 min, 2% B; 0.8–2.8 min, 2%–70% B; 2.8–5.6 min, 70%–90% B; 5.6–6.4 min, 90%–100% B; 6.4–8.0 min, 100% B; 8.0–8.1 min, 100%–2% B; and 8.1–10.0 min, 2% B. Metabolite detection was performed using a Q-Exactive high-resolution tandem mass spectrometer in both positive and negative ionization modes. Full-scan MS spectra (70–1050 m/z) were acquired at a resolution of 70,000, with an AGC target of 3×10^6^ and maximum injection time of 100 ms. MS/MS spectra were collected at a resolution of 17,500, with an AGC target of 1×10^5^ and maximum injection time of 80 ms. A quality control (QC) sample, pooled from all samples, was injected after every 10 experimental runs to monitor instrument stability. Statistical analysis was performed using Student’s t-test to compare metabolite levels between groups, with false discovery rate (FDR) correction for multiple comparisons using Benjamini–Hochberg method. Supervised partial least squares-discriminant analysis (PLS-DA) implemented in metaX was employed to identify group-discriminant metabolites, using a variable importance in projection (VIP) threshold of 1.0. Metabolites were considered significantly altered if they exhibited a fold change > 1.5, a Q-value < 0.05, and a VIP > 1. Functional enrichment analysis of significantly differential metabolites was further conducted using the OmicStudio platform (https://www.omicstudio.cn/tool).

***FMT:*** To investigate whether the protective effects of ICT on cognitive dysfunction were mediated by the gut microbiota, an FMT experiment was performed. All mice first received an ABX in their drinking water for 7 days to deplete gut microbiota and establish a pseudo germ-free state. The ABX solution contained ampicillin (1 g/L), vancomycin (1 g/L), neomycin sulfate (1 g/L), and metronidazole (1 g/L), and was replaced every two days to maintain potency. Fresh fecal samples were collected from donor mice 48 hours after the final ICT (20 mg/kg) gavage, following a 28-day treatment regimen. Each day, 1 g of feces was suspended in 5 mL of sterile distilled water, homogenized, and centrifuged at 1000 ×g for 10 min at 4 ℃. The supernatant was filtered through a 200-mesh sieve to obtain a bacterial suspension. Starting from the initiation of PFOS exposure, pseudo-germ-free recipient mice were administered 0.2 mL of the suspension via oral gavage daily for 28 consecutive days.

***Western blot (WB):*** Ileum tissue samples were homogenized in RIPA lysis buffer containing 1% protease inhibitor cocktail. The homogenates were centrifuged at 15,000 × g for 15 min at 4 ℃ to remove insoluble debris. The supernatant was collected, and total protein concentration was determined using a BCA protein assay kit. Proteins (10 μg per lane) were separated by SDS-PAGE on 6–12% gels and subsequently transferred to PVDF membranes via electroblotting. The membranes were blocked with 5% non-fat milk in TBST for 2 h at room temperature to minimize non-specific binding. Then, the membranes were incubated overnight at 4 ℃ with the following primary antibodies against Claudin-1 (1:1000), Occludin (1:1000), ZO-1 (1:1000), as well as *β*-actin (1:5000), *β*-tubulin (1:5000), which served as internal control. After washing, the membranes were probed with HRP-conjugated secondary antibodies for 40 min at room temperature. Protein bands were visualized using an ECL Western blot detection kit (MA0186, Merck Millipore, China) and imaged with a ChemiDoc MP Imaging System (Bio-Rad Laboratories, USA). Band intensities were quantified with Image Lab software, and the expression levels of tight junction proteins were normalized to *β*-actin and *β*-tubulin.

**
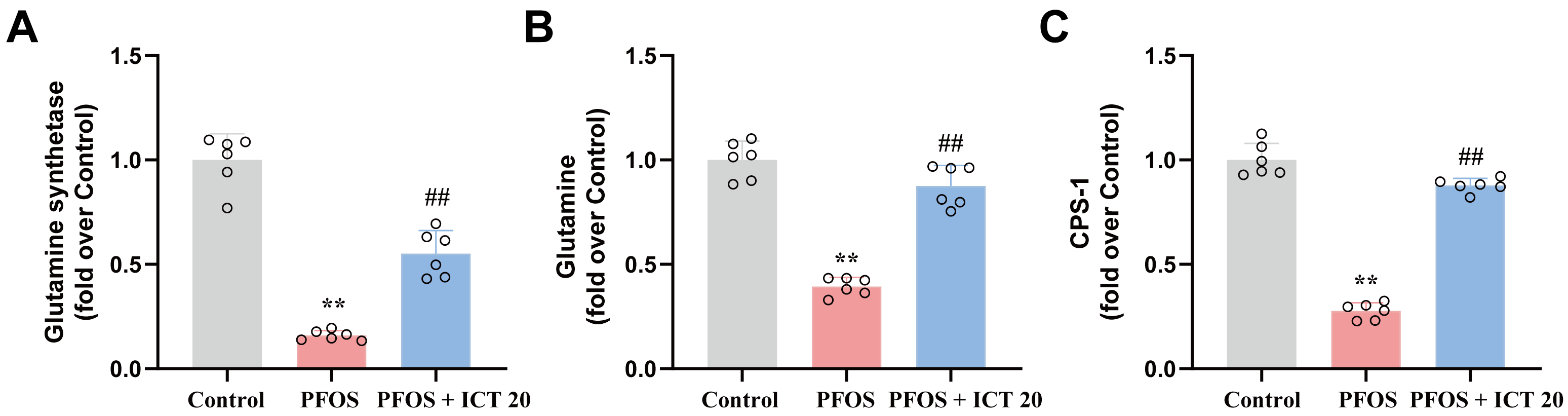
**

**Figure S1. ICT ameliorates brain ammonia metabolism by enhancing the activity of key detoxification enzymes.** (A)​Cerebral glutamine synthetase (GS) levels measured by ELISA (*n* = 6). (B) Cerebral glutamine (Gln) levels measured by ELISA (*n* = 6). (C) Cerebral carbamoyl-phosphate synthase 1 (CPS-1) levels measured by ELISA (*n* = 6). The data were presented as the mean ± SEM. ^**^*P* < 0.01 *vs.* Control group; ^#^*P* < 0.05, ^##^*P* < 0.01 *vs.* PFOS group.

**
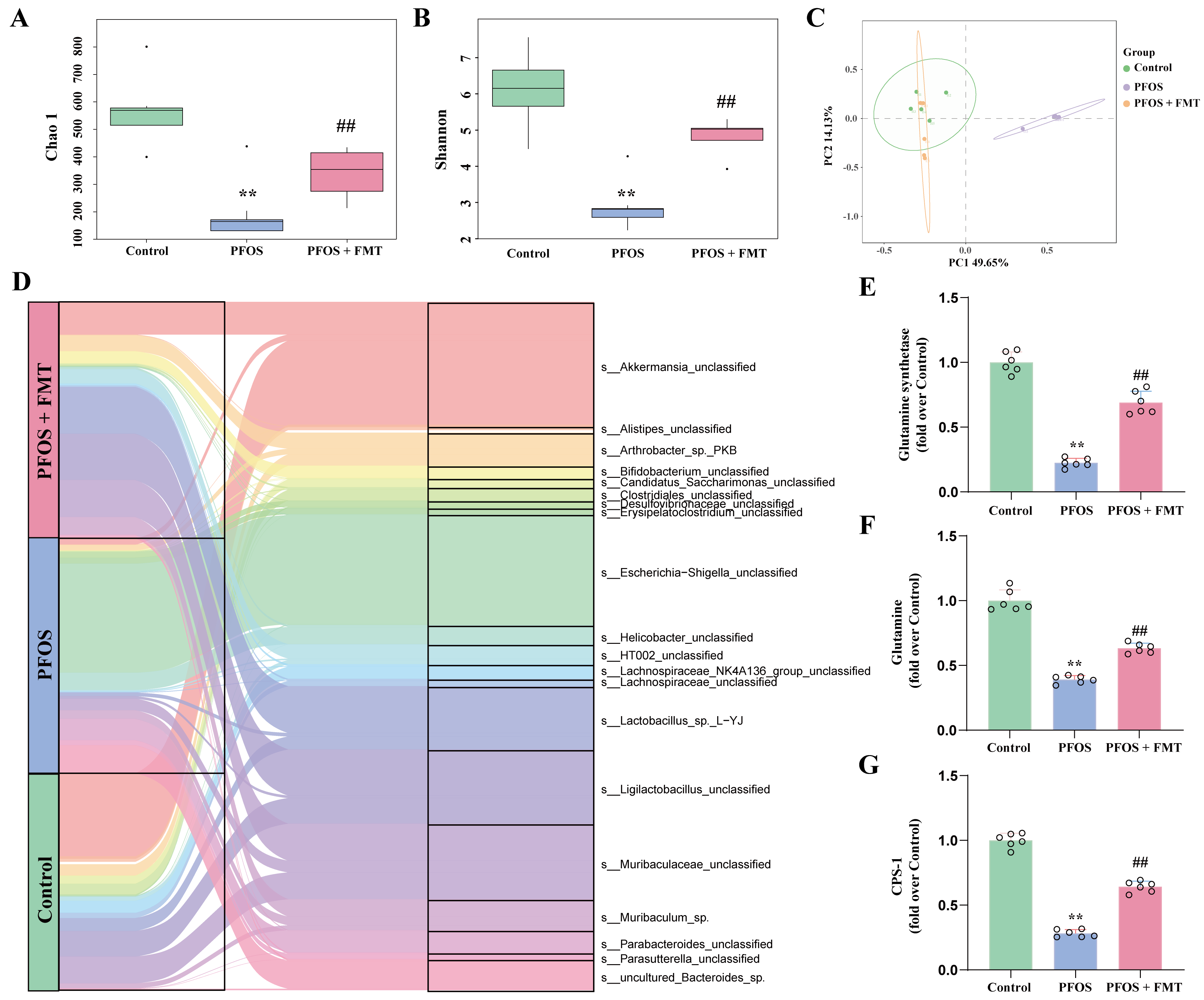
**

**Figure S2. FMT from ICT-treated donors partially restores PFOS-induced dysbiosis and ameliorates brain ammonia metabolism by enhancing the activity of key detoxification enzymes.**​(A)​ Chao1 (*n* = 5). (B)​Shannon (*n* = 5). (C) Principal coordinate analysis (PCoA) of β-diversity based on Bray–Curtis distance, illustrating structural shifts in microbial communities among groups. (D) Sankey diagram visualizing taxonomic composition and abundance dynamics across groups. (E) Cerebral GS levels measured by ELISA (*n* = 6). (F) Cerebral Gln levels measured by ELISA (*n* = 6). (G) Cerebral CPS-1 levels measured by ELISA (*n* = 6). The data were presented as the mean ± SEM. ^**^*P* < 0.01 *vs.* Control group; ^#^*P* < 0.05, ^##^*P* < 0.01 *vs.* PFOS group.

**
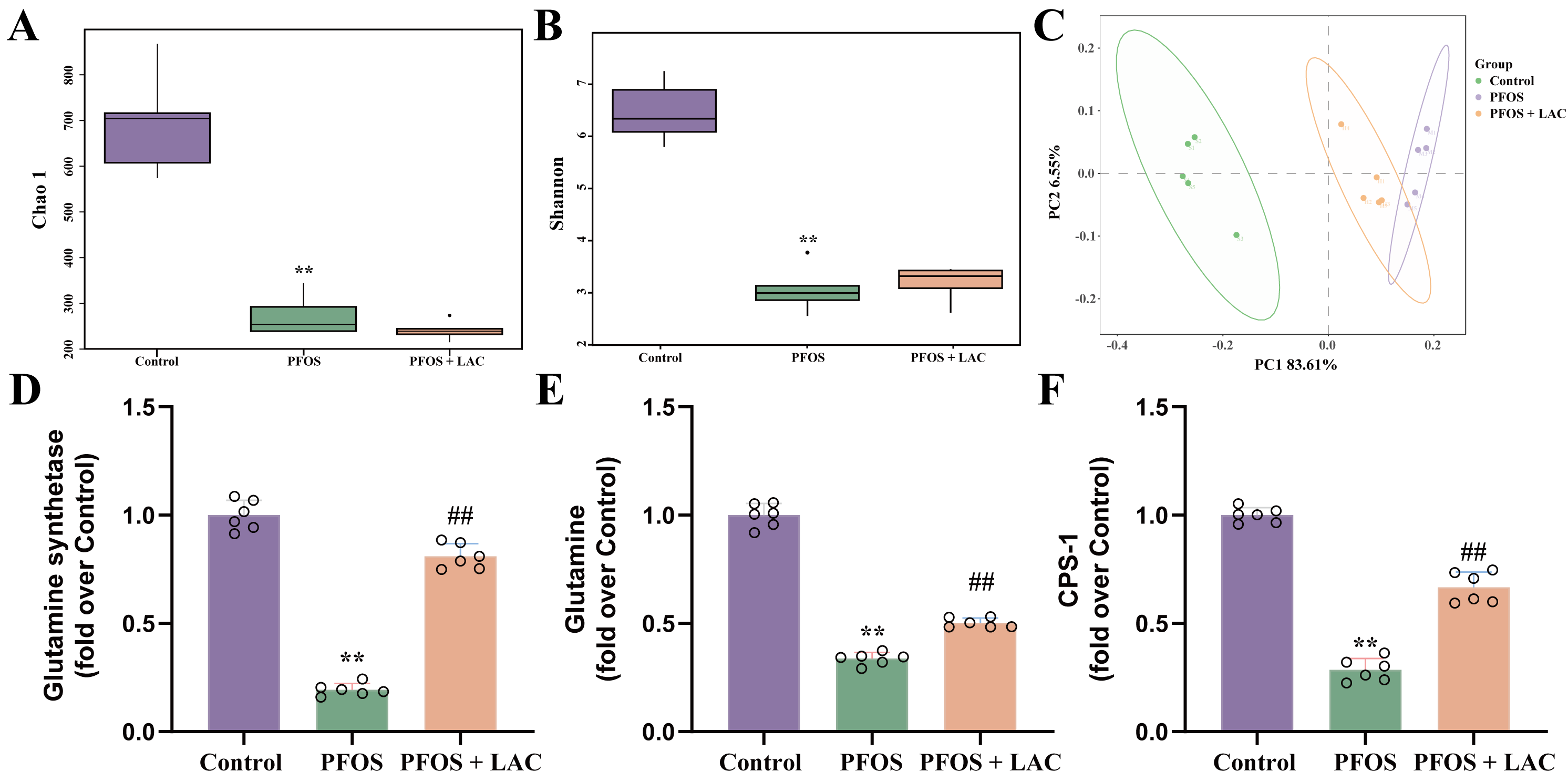
**

**Figure S3. LAC alleviates PFOS-induced impairments in ammonia metabolism without altering gut microbiota structure.​** (A) Chao1 (*n* = 5). (B) Shannon (*n* = 5). (C) PCoA of β-diversity based on Bray–Curtis distance. (D) Cerebral GS levels measured by ELISA (*n* = 6). (E) Cerebral Gln levels measured by ELISA (*n* = 6). (F)​Cerebral CPS-1 levels measured by ELISA (*n* = 6). The data were presented as the mean ± SEM. ^**^*P* < 0.01 *vs.* Control group; ^#^*P* < 0.05, ^##^*P* < 0.01 *vs.* PFOS group.

**References**

[1] Gao J M, Zhang X, Shu G T, Chen N N, Zhang J Y, Xu F, Li F, Liu Y G, Wei Y, He Y Q, Shi J S, Gong Q H. Trilobatin rescues cognitive impairment of Alzheimer's disease by targeting HMGB1 through mediating SIRT3/SOD2 signaling pathway[J]. Acta Pharmacol Sin, 2022, 43(10): 2482-2494.

[2] Wu X, Wei J, Ran W, Liu D, Yi Y, Gong M, Liu X, Gong Q, Li H, Gao J. The Gut Microbiota-Xanthurenic Acid-Aromatic Hydrocarbon Receptor Axis Mediates the Anticolitic Effects of Trilobatin[J]. Adv Sci (Weinh), 2025, 12(10): e2412234.
